# Hypoventilation and sleep hypercapnia in a case of congenital variant-like Rett syndrome

**DOI:** 10.1186/s13052-022-01359-7

**Published:** 2022-09-07

**Authors:** Sergio Ghirardo, Letizia Sabatini, Alessandro Onofri, Maria Beatrice Chiarini Testa, Maria Giovanna Paglietti, Daria Diodato, Lorena Travaglini, Fabrizia Stregapede, Marta Luisa Ciofi degli Atti, Claudio Cherchi, Renato Cutrera

**Affiliations:** 1 Pediatric Pulmonology & Respiratory Intermediate Care Unit, Academic Department of Pediatrics; Clinical, management and technology innovation research area, Medical Direction, Bambino Gesù Children’s Hospital IRCCS, Piazza S. Onofrio 4, 00165 Rome, Italy; 2 University Department of Pediatrics, Bambino Gesù Children’s Hospital IRCCS, University of Rome Tor Vergata, Rome, Italy; 3 Department of Neurosciences, Unit of Muscular and Neurodegenerative Disorders, Laboratory of Molecular Medicine, Bambino Gesù Children’s Hospital, IRCCS, Rome, Italy; 4 Clinical, management and technology innovation research area, Medical Direction, Bambino Gesù Children’s Hospital IRCCS, Rome, Italy

**Keywords:** Rett syndrome, Congenital variant, Hypotonia, Hypoventilation, Hypercapnia, Non-invasive ventilation

## Abstract

**Background:**

Breathing disturbances are often a primary clinical concern especially during wakefulness of the classic form of Rett syndrome, but data for atypical forms are lacking.

**Case presentation:**

We report the case of a 20-month-old female affected by Rett syndrome with congenital variant-like onset, characterized by severe hypotonia and neurodevelopment impairment. She presented hypoventilation, persistent periodic breathing, and sustained desaturation during sleep, without obstructive or mixed events. Pulse oximetry and capnography during wakefulness were strictly normal. To the best of our knowledge, this is the first case of a patient affected by a congenital variant of Rett syndrome presenting sleep hypercapnia. Hypotonia may play a major role in the genesis of hypoventilation and hypoxemia in our patient. Non-invasive ventilation led to quality-of-life improvements.

**Conclusions:**

Thus, we suggest screening patients with congenital-like Rett syndrome through transcutaneous bedtime carbon dioxide and oxygen monitoring. Moreover, assisted control mode was a breakthrough to achieve adequate ventilation in our case.

## Background

Rett syndrome (RS) (OMIM #312750) is a severe X-linked dominant neurodevelopmental disorder, affecting one baby every 10.000 live-born, almost exclusively female. The congenital variant of Rett syndrome (CRS) accounts for just 5–7% of all cases, and the clinical picture is characterized by severe hypotonia, often present at birth, and neurodevelopmental delay since the very first few months. Classic RS leads to remarkably breathing instability, usually even more pronounced during wakefulness [1]. We report a 20-month-old female presenting a congenital variant-like Rett syndrome, who showed central apneas, periodic breathing, and persistent hypercapnia only during sleep-time without obstructive apnea. Such findings were never reported before in a child affected by Rett syndrome. We present the ventilatory and clinical management of this unique case.

## Case presentation

The patient is a full-term (39 gestational weeks) third born Caucasian girl from Italy. The delivery was eutocial with no perinatal issues. Pregnancy was unremarkable, with no recall of reduced foetal movements. Birth weight was 2,93 kg (30° centile (pc), length 47 cm, head circumference 33 cm. Apgar index was 8 at one and 5 min. At birth, she presented with hypotonia. Standard metabolic screening performed as part of the routine care to newborns in Italy was negative. Family history was unremarkable. She was admitted due to hyperbilirubinemia and a urinary tract infection on the fifth day of life. Central hypotonia was remarkable. Full oral feeding was possible, however she presented reduced weight gain and recurrent emesis. Fundus oculi, cranial and abdominal ultrasound were normal; cardiac ultrasound showed only the persistency of the foramen ovale. She tested negative for Angelman and Prader-Willi diseases. At the age of 2 months, brain magnetic resonance (MR) was reported unremarkable, and she was discharged shortly after.

At the age of 10 months, she came to our attention, the psychomotor delay had become evident, and she scored < 70 on the Developmental Profile-3 test. She did not properly control the head; she reached but did not grasp objects and fixed her gaze just for up to 3 min. The weight was 8.5 (47°pc), length 67 cm (6°pc) and head circumference was 44 cm (9°pc). CGH-array and extensive metabolic screening (blood lactate, ammonium, acetyl carnitine panel, urinary organic amino acids, plasma amino acids levels, tandem mass spectrometry on blood and Barry test, including lumbar puncture (liquor physical-chemical analysis, lactate, neurotransmitters within normal range), tested negative; evoked potentials (visual evoked potentials VEP, electroretinogram ERG, auditory evoked potentials AEP and brainstem auditory evoked potentials BAEPs) were within normal ranges. Intensive physiotherapy allowed her to eat semi-liquid foods safely, reach and grab objects with her hands and maintain ocular contact for several minutes. Despite efforts, axial hypotonia progressively worsened in the following months, and the hand’s grip remained poor. At the age of 16 months, she underwent brain MR showing a mild bilateral ventricle and subarachnoid spaces widening; brain MR spectroscopy was normal. Clinical exome analysis with Next Generation Sequencing (NGS) approach provided a diagnosis of Rett syndrome 3-months after identifying a de novo heterozygous truncating variant caused by an insertion c.396_397insA (p.Arg133fsTer2) in *MECP2* gene (MIM *300005). This variant was never reported in any publicly available human variation resources (i.e. dbSNP, 1000 Genomes, ExAC, gnomAD). After that, we performed polysomnography, showing a poorly-organized electrical activity and diffuse irritating signals at the electroencephalogram (EEG). Due to hypotonia, we performed an x-ray of the column, revealing extremely precocious scoliosis (T4-L1 Cobb angle 26°). PSG was performed using Somtè PSG (Compumedics, Australia). Cardiorespiratory data included airflow (nasal pressure transducer and oronasal thermistor if available), body position, body movements, thoracic and abdominal movements assessed by respiratory inductance belts, SpO2, and video recording. The electroencephalographic record was based on the international 10–20 system with electrodes in positions F1-A2, F2-A1, C3-A2, C4-A1, O1-A2, O2-A1, recording eye movements. Transcutaneous carbon dioxide pressure (PtcCO2) recording was performed simultaneously (SenTec Digital Monitor, SenTec Inc., Therwil, Switzerland). Scoring of respiratory events was performed by an experienced reader, according to the American Academy of sleep medicine (AASM) criteria: obstructive apnea was defined as the absence of nasal airflow with continued chest movements for at least two breaths. Central apnea was defined as the absence of nasal airflow with the interruption of respiratory effort lasting more than 20 seconds or associated with arousal and/or a 3% oxygen desaturation. Periodic breathing was defined as three or more episodes of central apnea lasting > 3 seconds each and separated by < 20 seconds of normal breathing. She spent 55% of her total sleep-time (TST) with peripheral oxygen saturation (SpO_2_) < 90%. Her oxygen desaturations index (ODI) was 13.9, defined as the number of desaturations > 3% per hour of TST. Periodic breathing (PB) accounted for 25% of TST matching criteria for persistent periodic breathing (PPB) [2]. The mean percutaneous carbon dioxide partial pressure (PtcCO_2_) during bedtime was 50 mmHg, and she spent 57% of the TST above this limit. Therefore, she fulfilled even the stricter paediatric criteria for hypoventilation based on persistent overnight hypercapnia. Obstructive events were virtually absent with a 0.2 of mixed-obstructive hypopnea apnoea index (MOHAI), defined as the number of such episodes per hour of TST. Following the clinical evidence of a huge difference in breathing patterns between daytime and bedtime, we performed measurements of SpO2 and PtcCO2 during wakefulness, which were strictly normal (mean PtcCO2 35 mmHg, range 34–36 mmHg). Therefore, the increase of PtcCO2 between wakefulness and sleep was greater than 10 mmHg and provided further confirmation of a purely sleep time hypercapnia.

Non-invasive positive pressure ventilation (NIV) in room air was started during bedtime. We set spontaneous timed ventilation with shrink spam to enhance adaptation inspiratory positive airway pressure (IPAP) 8 cmH_2_O, expiratory positive airway pressure (EPAP) 4 cmH_2_O, with a respiratory frequency of 23 breaths per minute (very close to the patient one). Such setting normalized SpO_2_ values with minimum SpO_2_ 92% and an improvement of ODI to 7.2 events/hour. Carbon dioxide even worsened with a mean PtcCO_2_ of 51.7 mmHg, and 100% of TST spent above 50 mmHg. The first attempt was to increase IPAP to 14, reaching a partial improvement of PtcCO_2_, lowering the percentage spent above 50 mmHg to 29% of TST. At the same time, ODI was reduced to 0.5 events/hour. Shifting ventilation mode to adaptive pressure controlled (APC) mode and slightly increasing IPAP to 16 cmH_2_O, we finally obtained PtcCO_2_ normalization (peak 49 mmHg). In the few following days, a slight improvement of the hypotonia and social interaction with the caregiver was noticed. We will perform brief hospitalization to perform sleep studies (capnography and polysomnography) together with neurological, neuromuscular, neurodevelopmental evaluations and physiotherapy assessment after 4 months and every 6 months after.

## Discussion and conclusions

Congenital variant of Rett syndrome, also known as Rolando variant, presents with congenital hypotonia, poor responsiveness and eye contact. Developmental delay becomes evident already during the first few months of life with social impairment and autistic behaviours, and no language development. Head circumference is normal at birth but head growth is reduced with progressive microcephaly. Gross and fine motricity is impaired early, epilepsy develops after the first year of life but EEG is usually poorly organized since early stages [3–6]. The association of such variant of Rett syndrome and the mutation of FOXG1 is becoming recognised as a specific entity called FOXG1 syndrome and we endorse this to avoid terminological overlaps [3]. As our patient presents all the clinical presentation of Rolando variant, we consider she has a congenital variant-like Rett syndrome. Moreover, it is becoming clear that various mutations of FOXG1 gene lead to a wide range of clinical manifestations that can completely differ from the congenital variant of Rett syndrome [7]. At the other side, some patients with congenital Rett syndrome-like features do not present FOXG1 mutations as in our case [8]. our patient, in particular, shows a mutation encoding for a stop codon with a likely deep impairment in the protein function. Such type of mutation fits with the particularly severe clinical picture of the patient and the neonatal onset in contrast with what was previously reported [9]. Rett syndrome-related breathing features, like hyperpnoea followed by breath-holding, Valsalva efforts, and periodic breathing, are easily identifiable during wakefulness. Such symptoms usually reduce or even abruptly disappear at sleep onset [1, 10]. Among the patients with Rett syndrome with pathological polygraph, nearly all manifest obstructive events during sleep, with a minority of central events associated with severe obstructive ones [11]. Literature reports several cases of patients with RS presenting hypoventilation, often alternated to hyperventilation during wakefulness [12, 13]. On the contrary, few data are reported about hypercapnia during sleep time in RS mentioning congenital variant or congenital variant-like [13, 14]. Our patient presents congenital variant-like Rett syndrome caused by a novel de novo mutation in *MECP2*. *MECP2* gene plays an important role in autonomic system regulation and its mutations are causative of Rett syndrome [15]. Animal models showed the importance of MECP2 for a correct sensitivity to CO2 of breathing centres, and its mutations may lead to the typical respiratory instability of RS [16], and possibly to hypoventilation as proved by animal models [17]. To the best of our knowledge, this is the first case reporting hypoxemia and persistent hypercapnia during sleep-time in a patient affected by a congenital form of RS. Patients with CRS are a peculiar cohort; they never walk autonomously and may acquire the ability to articulate a few simple words at best, usually showing severe mental retardation since the very first months of life. Only a few cases present epilepsy (the landmark of the classic form) at the diagnosis, but electrical activity alterations are nearly always identified at the EEG [8, 9, 18].

As previously stressed, the congenital variant is mainly characterized by hypotonia, that is severe enough to cause scoliosis within the very first years of life [5]. Therefore, in CRS, hypotonia may cause, per se, desaturation and hypercapbnia, as occur in other neuromuscular diseases of childhood, suggesting a different pathogenesis than classic RS [19]. The normal PtcCO2 values during waketime (when muscle strength is relatively increased) supports this suggestion [20, 21]. Of particular interest is the persistent periodic breathing of this patient. Breathing centres homeostasis is very fragile in RS due to *MECP2* mutation [16], with a consequent high gain loop causing periodic breathing. Such events may be boosted by repeated desaturations caused by CRS-related hypotonia [12, 13]. Therefore, although an ST mode of ventilation is usually preferred to maximize patient comfort, an assisted pressure-controlled mode could be necessary to overcome the lack of appropriate respiratory centres control. Providing adequate ventilation in such patients could contribute to the daytime quality of life and hypotonia improvement, as already reported for other diseases with hypotonia [22].

The diagnosis of neonatal hypotonia requires an integrated clinical and instrumental approach initially focused at excluding etiologically treatable conditions such as spinal motoneuron atrophy (SMA). NGS technique can have a key in the diagnosis of unsolved hypotonic conditions in early infancy.

This single case suggests the need for a very early polygraph or at least pulse oximetry plus capnography in each patient affected by the congenital variant of Rett syndrome. Broadly speaking, we suggest considering NGS techniques to reach tricky diagnosis such as Rett syndrome with congenital features in hypotonic patients.

## Data Availability

Not applicable (case report).

## Abbreviations

RS: Rett syndrome
CRS: Congenital Rett syndrome
MR: Magnetic resonance
VEP: Visual evoked potentials
ERG: Electroretinogram
AEP: Auditory evoked potentials
BAEPs: Brainstem auditory evoked potentials
NGS: Next generation sequencing
EEG: Electroencephalogram
TST: Total sleep time
SpO2: Peripheral oxygen saturation
ODI: Oxygen desaturation index
PB: Periodic breathing
PPB: Protracted periodic breathing
PtcCO2: Carbon dioxide partial pressure
MOHAI: Mixed obstructive hypopnea apnoea index
NIV: Non-invasive ventilation
EPAP: Expiratory positive airway pressure
IPAP: Inspiratory positive airway pressure
APC: Adaptive pressure controlled
